# Occurrence and Fate of Ultramicrobacteria in a Full-Scale Drinking Water Treatment Plant

**DOI:** 10.3389/fmicb.2018.02922

**Published:** 2018-12-05

**Authors:** Jie Liu, Renxin Zhao, Jiayu Zhang, Guijuan Zhang, Ke Yu, Xiaoyan Li, Bing Li

**Affiliations:** ^1^Guangdong Provincial Engineering Research Center for Urban Water Recycling and Environmental Safety, Graduate School at Shenzhen, Tsinghua University, Shenzhen, China; ^2^School of Environment and Energy, Shenzhen Graduate School, Peking University, Shenzhen, China

**Keywords:** ultramicrobacteria, drinking water treatment, microbial community, high-throughput sequencing, flow cytometry, 0.22-μm filtration, metabolic functions, genome streamlining

## Abstract

Ultramicrobacteria (UMB) are omnipresent and numerically dominate in freshwater, as microbes can present in drinking water systems, however, the UMB communities that occur and their removal behaviors remain poorly characterized in drinking water treatment plants (DWTPs). To gain insights into these issues, we profiled bacterial cell density, community structure and functions of UMB and their counterpart large bacteria (LB) using flow cytometry and filtration paired with 16S rRNA gene high-throughput sequencing in a full-scale DWTP. Contrary to the reduction of bacterial density and diversity, the proportion of UMB in the total bacteria community increased as the drinking water treatment process progressed, and biological activated carbon facilitated bacterial growth. Moreover, UMB were less diverse than LB, and their community structure and predicted functions were significantly different. In the DWTP, UMB indicator taxa were mainly affiliated with α/β/γ-*Proteobacteria, Deinococcus–Thermus, Firmicutes, Acidobacteria*, and *Dependentiae*. In particular, the exclusive clustering of UMB at the phylum level, e.g., *Parcubacteria, Elusimicrobia*, and *Saccharibacteria*, confirmed the fact that the ultra-small size of UMB is a naturally and evolutionarily conserved trait. Additionally, the streamlined genome could be connected to UMB, such as candidate phyla radiation (CPR) bacteria, following a symbiotic or parasitic lifestyle, which then leads to the observed high connectedness, i.e., non-random intra-taxa co-occurrence patterns within UMB. Functional prediction analysis revealed that environmental information processing and DNA replication and repair likely contribute to the higher resistance of UMB to drinking water treatment processes in comparison to LB. Overall, the study provides valuable insights into the occurrence and fate of UMB regarding community structure, phylogenetic characteristics and potential functions in a full-scale DWTP, and it is a useful reference for beneficial manipulation of the drinking water microbiome.

## Introduction

Bacteria with an ultra-small cell size are omnipresent and numerically dominate in some natural aquatic ecosystems, including oceans, rivers, lakes, and groundwater (Cavicchioli and Ostrowski, 2003; Duda et al., 2012; Luef et al., 2015; Neuenschwander et al., 2015; Proctor et al., 2018). These small bacteria are known by a variety of terms such as “ultramicrobacteria (UMB),” “ultra-small bacteria,” “ultramicrocells,” “nanobacteria” or “low nucleic acid content (LNA) bacteria” (Cavicchioli and Ostrowski, 2003; Duda et al., 2012; Proctor et al., 2018). The UMB have small genomes (0.58–3.2 Mb) and an essentially constant cell volume of less than 0.1 μm^3^, independent of culture conditions, such as growth stages and nutrient concentrations (Cavicchioli and Ostrowski, 2003; Duda et al., 2012). UMB are found within phylogenetically distinct groups, including the *Actinobacteria, Proteobacteria, Spirochaetes* and the *Cytophaga- Flavobacterium- Bacteroides* branch (Hahn, 2004; Hahn et al., 2004; Miteva and Brenchley, 2005; Duda et al., 2012; Proctor et al., 2018).

The tiny cell size of UMB provides a larger surface-to-volume ratio, allowing more efficient uptake of nutrients from oligotrophic environments (Giovannoni et al., 2005; Duda et al., 2012) and protection against grazing pressure (Miteva and Brenchley, 2005; Williams et al., 2009). Furthermore, the streamlined genome of UMB optimizes and simplifies bacterial metabolism (Giovannoni et al., 2005; Williams et al., 2009). This minimizes maintenance requirements (e.g., phosphorus and nitrogen) for DNA synthesis and cell growth, permits efficient nutrient utilization and facilitates growth in nutrient-depleted conditions (Giovannoni et al., 2005, 2014; Williams et al., 2009). In addition, UMB can simultaneously assimilate a mixture of substrates, allowing relatively faster growth under carbon/energy-limited conditions (Egli, 2010). These versatile metabolic properties contribute to the adaptive growth of UMB at low concentrations of nutrients and explains their wide distribution from aquatic biotopes to terrestrial ecosystems (Cavicchioli and Ostrowski, 2003; Moran et al., 2015), even extreme habitats such as permafrost soils, ice sheet and thermal swamp moss (Loveland-Curtze et al., 2010; Suzina et al., 2015).

Biodiversity, population dynamics and functional activities of bacterial communities in drinking water treatment plants (DWTPs) are well documented (Ramseier et al., 2011; Besmer and Hammes, 2016; Zhang et al., 2017; Oh et al., 2018). The filtration process of DWTPs, including rapid sand filtration and biological granular activated carbon filtration (BAC) was designed to remove suspended particles, bacteria and dissolved organic matter through microbial degradation (Boon et al., 2011; Velten et al., 2011; Pinto et al., 2012; Basu et al., 2016). As a hygienic barrier, a disinfection step, e.g., ozonation, chlorination, chloramination, or ultraviolet (UV) treatment, was used to inactivate primary and opportunistic pathogenic bacteria to guarantee microbial quality in drinking water distribution systems (Gomez-Alvarez et al., 2012; Chiao et al., 2014; Inkinen et al., 2018). To date, it has been recognized that UMB are not simply dormant members of the microbial population, but play important functions in biogeochemical cycling of organic and inorganic matters (Cavicchioli and Ostrowski, 2003; Williams et al., 2009; Duda et al., 2012). As the heterotrophic and autotrophic bacteria are able to eliminate contaminants in DWTPs (Oh et al., 2018), UMB would stimulate the formation of a biofilm with a high metabolic biodiversity and thus can remediate polluted aquifers (Ross et al., 2001). It was also reported that UMB were capable of anaerobic dechlorination of polychlorinated biphenyls (May et al., 2008) and mineralizing herbicide at low concentration (Gözdereliler et al., 2013). However, bacterial growth in drinking water can lead to water quality decline and health risks, e.g., pathogen occurrence (Hou et al., 2018; Oh et al., 2018). Previous studies have shown that UMB possess extreme resistance mechanisms to cross-protect against various environmental stresses, such as high-intensity heat shocks, UV, viral infection, antibiotics and oxidative stress, e.g., treatments in H_2_O_2_ (Eguchi et al., 1996; Cavicchioli and Ostrowski, 2003). Ramseier et al. (2011) found that some UMB cell membranes exhibited lower damage reactivity than large bacteria (LB) during disinfection treatments using chlorine dioxide and permanganate. Furthermore, UMB can escape the 0.2-μm microfiltration trapping. Wang et al. (2008b) found that more than 10% of indigenous bacteria could pass through 0.22-μm pore size industrial-scale cartridge filtration units in groundwater aquifers. Recent studies revealed that UMB, termed LNA bacteria in flow cytometry (FCM), are more abundant than LB in drinking water (Liu et al., 2016, 2017b). Moreover, UMB may use a dormancy strategy for survival even under unfavorable growth conditions (Roesel and Grossart, 2012). It is therefore crucial to comprehensively investigate UMB community structure and their removal behaviors in DWTPs, and this could contribute to the beneficial manipulation of the drinking water microbiome.

In the current study, we aimed to test the hypothesis that UMB were more recalcitrant to removal than their counterpart LB in DWTPs. The specific objectives of the study were to: (1) profile the changes of bacterial cell density and community structure of UMB and LB in DWTPs via FCM and filtration paired with 16S rRNA gene sequencing; (2) identify indicator taxa, phylogenetic difference and fate of UMB and LB in DWTPs; and (3) predict and compare potential functional and metabolic capabilities of UMB and LB.

## Materials and Methods

### Water Sampling

Water samples were taken from a DWTP located in Guangdong province, China. The treatment processes of this DWTP comprised pre-ozonation, flocculation-sedimentation, rapid sand filtration, ozonation, biological granular activated carbon filtration and chlorination with a daily treatment capacity of 520,000 m^3^. The raw water (RW) and effluent of each process unit, i.e., flocculation-sedimentation effluent (FS), rapid sand filtration effluent (RSF), biological granular activated carbon filtration effluent (BAC), and chlorination water (CW), were sampled in 2017 on April 21 (DWTP1), May 6 (DWTP2), and May 24 (DWTP3), respectively. Volumes per sampling units ranged from 10 to 50 L depending on the pre-measured cell density of bacteria in the respective drinking water treatment units. Each filtration step was performed using 5–10 pieces of 47-mm filter, and filters in each step were pooled and then used to extract genomic DNA. It should be noted that the BAC tanks were in a state of maintenance on May 6. Therefore, we cannot collect the BAC effluent sample at that time. Residual chlorine was quenched using Na_2_S_2_O_3_ immediately during sampling. Water samples were kept in a 4°C ice box and then transported to the laboratory and processed for FCM within 3 h.

### Separation of Large Bacteria and Ultramicrobacteria

A two-step filtration was used to obtain LB and UMB (Luef et al., 2015). The original water sample was initially filtered through 0.22-μm polyvinylidene fluoride membranes (47 mm, Millipore Cork, Ireland) using sterilized filtration units (Nalgene, Thermo Fisher Scientific, United States) mounted on sterilized glass bottles. Bacteria in the 0.22-μm filtrates were subsequently refiltered through 0.10-μm polycarbonate membranes (47 mm, Millipore). Bacterial cell density of the original water samples and 0.22/0.10-μm filtrates was measured immediately via FCM. Membranes collected from the 0.22-μm (LB, captured on 0.22-μm filter) and 0.10-μm (UMB, passing 0.22-μm filter and captured on 0.10-μm filter) filtration steps were stored at -80°C for later DNA extraction.

Bacterial cell density (cells/mL) was measured with FCM in the original water samples (*CD*_original_) and 0.22-μm filtrates (*CD*_UMB_), where LB cell density (*CD*_LB_) was calculated as follows (Eq. 1):

(1) $$CD_{\text{LB}}=CD_{\text{original}}-CD_{\text{UMB}}$$

The UMB proportion (*UMB*%) was expressed as follows (Eq. 2).

(2) $$UMB\%=(CD_{\text{UMB}}/CD_{\text{original}})\times 100\%$$

### Flow Cytometry

Flow cytometric measurements were carried out as described previously (Liu et al., 2017a). One milliliter of water sample was stained with 10 μL/mL SYBR Green I (1:100 dilutions in dimethyl sulfoxide as the working solution; Invitrogen, United States), and incubated in the dark for 15 min at room temperature. FCM then was performed at a flow rate of 60 μL/min using a BD FACSCalibur system (BD Biosciences, San Jose, CA, United States) equipped with a 15-mW air-cooled argon laser, emitting at a fixed wavelength of 488 nm. All data were analyzed with the BD CellQuest Pro software (BD Biosciences). Green fluorescence was collected in the FL1 channel (520 ± 20 nm) and bacterial cell density was counted through the gating on a two-parameter dot-plot of green fluorescence against side scatter. Milli-Q cell-free water was used as the blank samples and sheath fluid as well in FCM. Positive signals of bacterial cell was separated from instrument noise or sample background by electronic gating with BD CellQuest Pro software (BD Biosciences). All samples were collected as logarithmic signals and were triggered on the green fluorescence channel. The coefficient of variation on FCM measurement for replicates should be below 2%, and all samples were measured in triplicate.

### 16S rRNA Gene Sequencing With Illumina HiSeq 2500 Platform

Genomic DNA was extracted from LB and UMB, respectively, retained on sterile 0.22-μm and 0.10-μm membranes using a FastDNA SPIN Kit for Soil (MP Biomedicals, United States) and following the manual protocol. Additionally, three sterilized filter were used as the blank samples, i.e., the negative control in DNA extraction and PCR amplification. The 16S rRNA V4 region sequences (∼265 nucleotides) were amplified using primers F515 (5′-GTG CCA GCM GCC GCG GTA A-3′) and R806 (5′-GGA CTA CHV GGG TWT CTA AT-3′) (Ma et al., 2017) and PrimeSTAR HS DNA Polymerase (Takara Biomedical Technology, Kusatsu, Japan). The amplification program was as follows: 95°C for 5 min followed by 30 cycles of 95°C for 30 s, 60°C for 30 s and 72°C for 1 min, and a final extension at 72°C for 10 min. PCR assays were conducted in triplicate to minimize variation during amplification, then the PCR triplicates were pooled and sequenced as one sample. PCR products were purified with TaKara miniBEST DNA Fragment Purification Kit (Takara Biomedical Technology) and the concentration was measured by NanoDrop One (Thermo Scientific, United States). The PCR product concentration of the blank samples was below the detection limit. All purified PCR amplicons were mixed together to achieve equal mass concentration and were sequenced on an Illumina HiSeq 2500 PE250 (Novogene, Tianjin, China). Sequences were analyzed using Mothur software v. 1.39.5 as described in Kozich et al. (2013). Chimeras were checked with Chimera.uchime and sequencing depth was finally normalized to 17,000 sequences per sample. The 16S rRNA gene sequences were clustered into operational taxonomic units (OTUs) based on a similarity threshold of 97%. Singleton OTUs were removed and excluded for the following analysis. Taxonomic annotation of representative OTU sequences was conducted using BLASTn search against the SILVA v132 reference database (Quast et al., 2013).

### Microbial Community and Co-occurrence Network Analysis

Rarified OTU tables were used to generate α- and β- diversity metrics. Species richness was the number of unique OTUs in the sample, and Shannon diversity index was calculated as the community α-diversity by R software using vegan package (Oksanen et al., 2018). Non-metric multidimensional scaling (NMDS) analysis was conducted to visualize community similarities using the Bray–Curtis dissimilarity as β-diversity. Adonis analysis was used to quantify the relative contribution of each factor (i.e., membrane pore size, treatment process units and each individual sampling) to the variation of community structure by vegan package. The linear discriminant analysis effect size (LEfSe) method was employed to discover specific indicator taxa for LB and UMB [threshold: logarithmic linear discriminant analysis (LDA) scores > 2 and Kruskal–Wallis test *P*-value < 0.05] (Segata et al., 2011). Those OTUs unassigned to indicator taxa were categorized into the “normal” taxa. To further visualize the differentiation of LB and UMB indicator taxa on the community composition, a taxonomic alike-tree was constructed at different classification levels using igraph package (Csardi and Nepusz, 2006). A phylogenetic tree was constructed using representative sequences of indicator taxa OTUs via the maximum likelihood method in MEGA 7, and then displayed via iTOL (Letunic and Bork, 2016).

A co-occurrence network analysis was performed for indicator taxa OTUs of LB and UMB associated with normal taxa OTUs according to the previous study (Ju and Zhang, 2015). Topological properties, including average weighted degree, network diameter, average path length, clustering coefficient and modularity, were calculated to describe the complex pattern of interrelationships among microbial taxa. The observed incidence (O%) and random incidence (R%) of co-occurrence patterns between taxa were statistically checked, and the degree of a lack of agreement between O% and R% (O/R ratio) was used as a benchmark of the non-randomness of observed co-occurrence patterns in bacterial communities.

### Microbial Functional and Metabolic Prediction

The potential functional and metabolic capacities of OTUs were predicted based on the SILVA123 and Kyoto Encyclopedia for Genes and Genomes (KEGG) database using the Tax4fun package (Asshauer et al., 2015). The taxonomic profile of KEGG was obtained by the SILVA-based OTUs normalized by the 16S rRNA gene copy number. The Procrustes analysis was used to compare the difference of functional and metabolic capacities between LB and UMB (Forsberg et al., 2014). Student’s *t*-tests were performed using the “ggpubr” package in R, and the *P*-values were adjusted with the “BH” (or “fdr”) method of Benjamini–Hochberg to control the false discovery rate. Significantly differential enzyme-encoding genes were extracted and four categories of stress-resistant related functions in UMB were further classified based on the KEGG database (Supplementary Table S1).

### Nucleotide Sequence Accession Numbers

Partial 16S rRNA gene sequences were deposited in the National Centre for Biotechnology Information (NCBI) Sequence Read Archive (SRA) under the accession number PRJNA481143.

## Results

### Changes of Cell Density and α-Diversity Along the Treatment Processes

**FIGURE 1:**
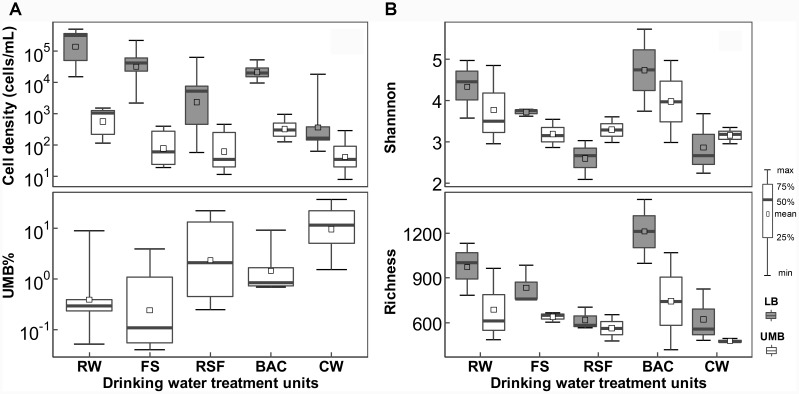
Changes in **(A)** cell density and **(B)** α-diversity for LB and UMB among treatment units of a DWTP. RW, raw water; FS, flocculation-sedimentation effluent; RSF, rapid sand filtration effluent; BAC, biological granular activated carbon filtration effluent; CW, chlorination water.

Both LB and UMB cell densities were significantly reduced along the treatment process in the DWTP [*P* < 0.01, by analysis of variance (ANOVA)], except after the BAC unit (LB: 0.94–5.38 × 10^4^ cells/mL; UMB: 1.22–9.68 × 10^2^ cells/mL). However, the UMB proportion (*UMB*%) significantly increased along treatment units of the DWTP from 0.05–9.05% to 1.52–37.4% (*P* < 0.05, ANOVA, Figure 1A). The rarefaction curve of the assigned sequences per sample and Good’s Coverage ranging from 0.88 to 0.96 demonstrated that the sequencing depth was sufficient for each sample (Supplementary Figure S1). After removal of singleton OTUs, a total of 3,033 OTUs were obtained, including 2,826 LB OTUs and 2,523 UMB OTUs, with 2,316 OTUs shared by both LB and UMB (Supplementary Figure S2A). Overall, UMB community, as well as core UMB microbiome shared throughout all treatment units, were less diverse than LB community and core LB microbiome, respectively, among treatment units with exception of RSF (Figure 1B and Supplementary Figure S2). Meanwhile, variation in Shannon diversity index and richness of UMB was also less than that of LB among treatment units (*P* > 0.05, ANOVA). The LB α-diversity including Shannon index and richness decreased along the treatment units in DWTPs (*P* < 0.05, ANOVA), with the exception of the BAC. The BAC samples showed a complete reversal of the previous reduction in Shannon and richness index, indicating the highest diversities in the entire drinking water treatment units.

### Variance of β-Diversity and Community Composition

**FIGURE 2:**
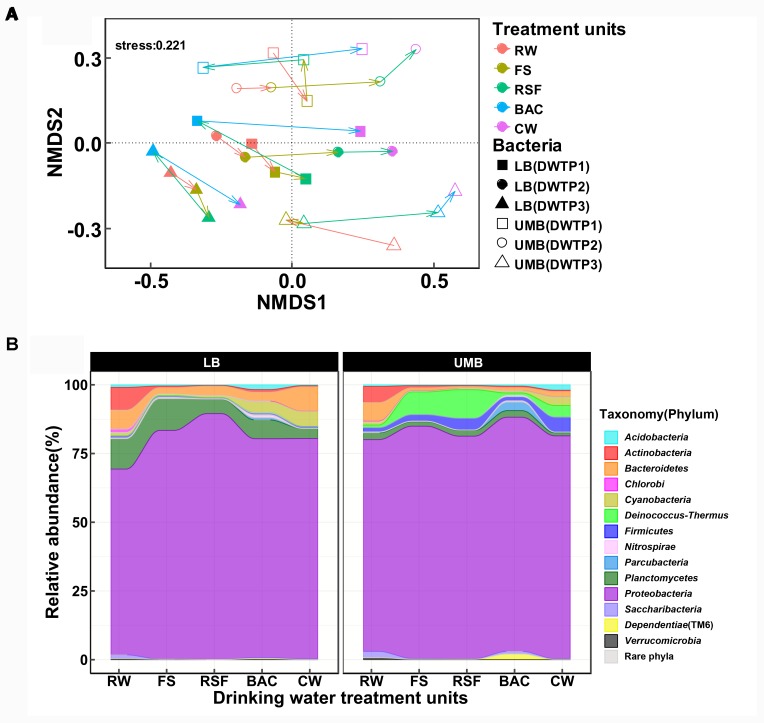
**(A)** Non-metric multidimensional scaling analysis (Bray–Curtis dissimilarity) for community structure of LB and UMB at the OTU level (97% similarity). **(B)** Variance of bacterial community composition of LB and UMB at the phylum level.

The NMDS analysis showed that LB and UMB are clearly separated for each sample and individual sampling, while treatment process-induced changes are clear, but relatively minor in comparison (Figure 2A). The effect of 0.22-μm size-separation, individual DWTP sampling and their interactions could explain 34.6% of all community variations (10.9%, 13.1%, and 10.6%, respectively, Adonis, *P* < 0.001). Meanwhile, the treatment processes considerably affected bacterial communities, accounting for 23.4% of all community variations (Adonis, *P* < 0.001). For the community composition, the *Proteobacteria* dominated both LB (67.6–89.4%) and UMB (77.0–85.2%) in the DWTP, followed by *Planctomycetes* (3.6–11.5%) and *Bacteroidetes* (2.3–8.9%) for LB, and *Deinococcus–Thermus* (0.8–8.3%) and *Firmicutes* (1.5–5.4%) for UMB (Figure 2B). With respect to changes of community compositions of both LB and UMB in DWTP, compared with the RW, the *Actinobacteria, Bacteroidetes*, and *Planctomycetes* in both LB and UMB decreased along treatment units, with the exception that LB *Bacteroidetes* in CW was higher than that of RW (Figure 2B). In contrast, the relative abundances of *Cyanobacteria, Deinococcus–Thermus*, and *Firmicutes* in both LB and UMB increased along the treatment units. The phylum *Proteobacteria* increased from RW to CW in LB, and slightly increased in UMB, while there was a reduction from BAC to CW in UMB, and a reduction from RSF to BAC in LB (Figure 2B).

### Occurrence of LB and UMB Indicator Taxa

**FIGURE 3:**
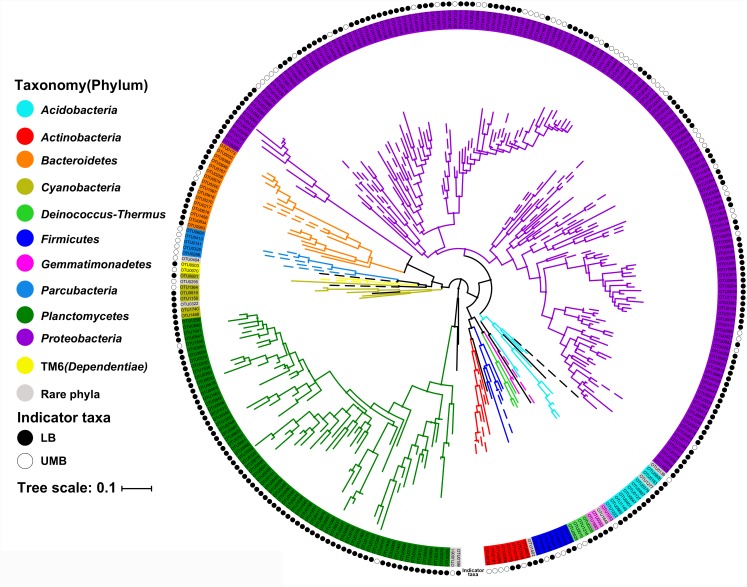
Maximum likelihood phylogenetic tree of LB and UMB indicator taxa. The branch color represents the taxonomy at the phylum level. Solid and open circles represent the LB and UMB indicator taxa, respectively. Bar, 10% sequence divergence.

The LEfSe algorithm can discover and explain differences in genomic features, including organisms, clades, OTUs, genes, or functions between two or more biological conditions in high-dimensional metagenomic data (Segata et al., 2011). Based on the differential OTUs abundance feature, 227 and 72 OTUs were, respectively, identified as the LB and UMB indicator taxa via the LEfSe algorithm (Supplementary Figure S3). LB indicator taxa accounted for 42.0% (ARA: the average of relative abundance of indicator taxa in all sample) of all LB communities (all 0.22-μm-filter-captured microbes) and UMB indicator taxa accounted for 37.4% of all UMB communities (all 0.1-μm-filter-captured microbes). These LB and UMB indicator taxa were phylogenetically located within 21 phyla (Figure 3). Both LB (ARA: 34.2%) and UMB (ARA: 27.9%) indicator taxa were mainly affiliated with phylum *Proteobacteria*, and several exclusive phyla were classified as UMB, i.e., *Parcubacteria* (OD1, ARA 0.25%), *Elusimicrobia* (ARA 0.10%) and *Saccharibacteria* (TM7, ARA 0.02%). The UMB indicator taxa also belonged to *Deinococcus–Thermus, Firmicutes, Acidobacteria*, and *Dependentiae* (TM6).

**FIGURE 4:**
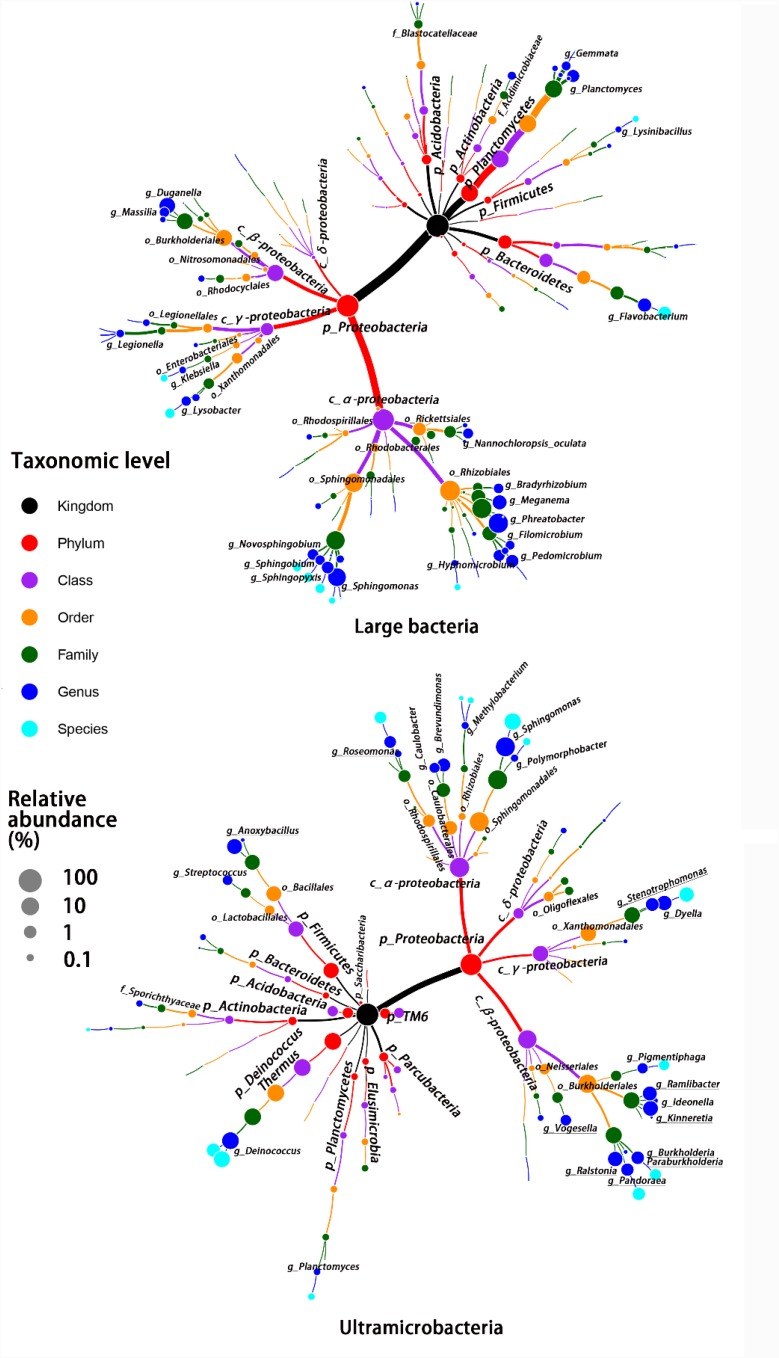
Taxonomic tree for LB and UMB indicator taxa composition at different taxon levels. Color and size of circles, respectively, represent the taxon level and average relative abundance of indicator taxa.

Despite there being no exclusivity observed in *Proteobacteria* phylum clusters, when taxa were considered on the genus level some exclusive genera were detected in UMB indicator taxa, such as *Kinneretia* (ARA 2.4%), *Ralstonia* (ARA 2.1%), *Dyella* (ARA 2.1%), *Ramlibacter* (ARA 1.8%), *Stenotrophomonas* (ARA 1.1%), *Burkholderia paraburkholderia* (ARA 1.1%), *Pandoraea* (ARA 1.0%), *Roseomonas* (ARA 0.8%), and *Vogesella* (ARA 0.5%) as well as *Anoxybacillus* (ARA 2.5%) belonging to the phylum *Firmicutes* (Figure 4). These genera mainly aligned to the family *Acetobacteraceae* in the α-*Proteobacteria*, the families *Neisseriaceae, Burkholderiaceae*, and *Comamonadaceae* in the β-*Proteobacteria*, and the family *Xanthomonadaceae* in the γ-*Proteobacteria.* With a few exceptions, LB and UMB indicator taxa overlapped within several branches, e.g., *Sphingomonas* in the α*-Proteobacteria* class and *Planctomyces* in the phylum *Planctomycetes*.

**FIGURE 5:**
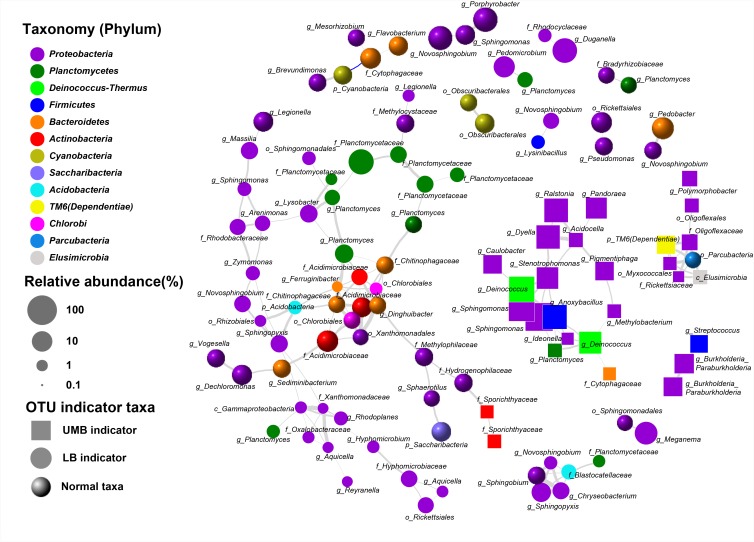
Co-occurrence of LB and UMB indicator taxa associated with normal taxa. Gray and blue curves represent significantly positive and negative correlation (*P* < 0.01). The thickness of the edges is proportional to the absolute value of Spearman’s correlation coefficient ranging from 0.8 to 1. The relative abundance cut-off values for the OTUs involved in network analysis were: indicator taxa OTUs > 0.02% and normal taxa OTUs > 0.1%. Each OTU was annotated into the lowest taxonomic rank.

Co-occurrence patterns of LB/UMB indicator taxa and normal OTUs were explored using network inference based on strong (*ρ* > 0.8 and *ρ* < -0.8) and significant (*P* < 0.01) correlations (Figure 5). The co-occurrence patterns consisted of 109 nodes and 161 edges. The average weighted degree or node connectivity was 2.51. The average network distance between all pairs of nodes, i.e., average path length, was 4.51 edges with a network diameter of 12 edges. The clustering coefficient (that is, how nodes are embedded in their neighborhood and, thus, the degree to which they tend to cluster together) was 0.48 and the modularity index was 0.81. Structural and statistical analyses demonstrated that non-random co-occurrence patterns were evident within the intra indicators and the intra phylum (Figure 5). For the OTUs from the intra indicator taxa, LB–LB (55 OTUs), UMB–UMB (43 OTUs), and normal–normal (33 OTUs) tended to co-occur much more (81.4%) than expected under random association (33.9%), despite their different degrees of intra-type co-occurrence (as reflected by ‘O/R ratio,’ O/R_LB-LB_: 2.0, O/R_UMB-UMB_: 4.2, O/R_normal-normal_: 1.9). Among the phyla, *Proteobacteria* had the highest non-random (O% = 36.4%) and random (R% = 37.9%) co-occurrence incidences (O/R = 0.96). *Actinobacteria, Bacteroidetes*, and *Planctomycetes* showed higher possibilities of non-random co-occurrence than of random positive association (O/R = 11.0, 5.1, and 2.8, respectively). Moreover, higher non-random co-occurrence incidences were also observed for the OTUs from inter phyla (Figure 5). For example, higher incidences of co-occurrence than expected by chance were observed between OTUs from *Proteobacteria* and other phyla, including *Acidobacteria, Deinococcus–Thermus, Elusimicrobia, Parcubacteria*, and TM6 (*Dependentiae*) (O% > 1.0%, O/R > 1.5).

### The Fate of Individual Indicator Taxa in the DWTP

**FIGURE 6:**
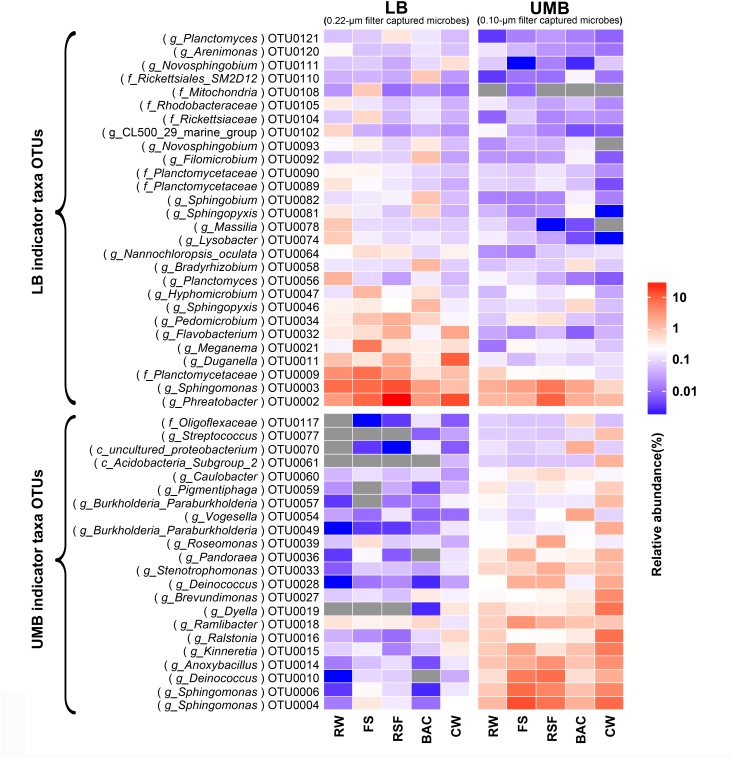
The fate of LB and UMB indicator taxa OTUs in the treatment units of a DWTP. Indicator taxa OTUs with a relative abundance more than 0.1% are shown, and each OTU is annotated into the lowest taxonomic rank.

Compared with the RW, the total relative abundance of UMB indicator taxa in all UMB communities (almost all UMB indicator taxa OTUs) increased along the following treatment units (FS, RSF, and CW) with the exception of BAC, where the total relative abundance decreased to a level similar to RW, i.e., 13.0% (RW) – 47.4% (FS) – 38.0% (RSF) – 20.3% (BAC) – 62.6% (CW) (ANOVA, *P* < 0.05) (Figure 6 and Supplementary Figure S4). For LB indicator taxa, the changes of total relative abundance of LB indicator taxa ranged as follows: 31.8% (RW) – 46.9% (FS) – 61.7% (RSF) – 27.4% (BAC) – 37.5% (CW). In comparison to UMB, the variation of LB indicator taxa were mainly attributed to OTU0002 (*Phreatobacter*: α-*Proteobacteria*), OTU0011 (*Duganella*: β-*Proteobacteria*) and OTU0032 (*Flavobacterium*: *Bacteroidetes*), and the remaining major LB indicator taxa gradually reduced along the treatment process in the DWTP. Interestingly, the total relative abundance of both LB and UMB indicator taxa did not show an increase accompanying the increasing cell density and biodiversity in the BAC (Figure 1), but decreased to similar levels as those in the RW (Figure 6 and Supplementary Figure S4).

### Prediction of Functional and Metabolic Capacities

**FIGURE 7:**
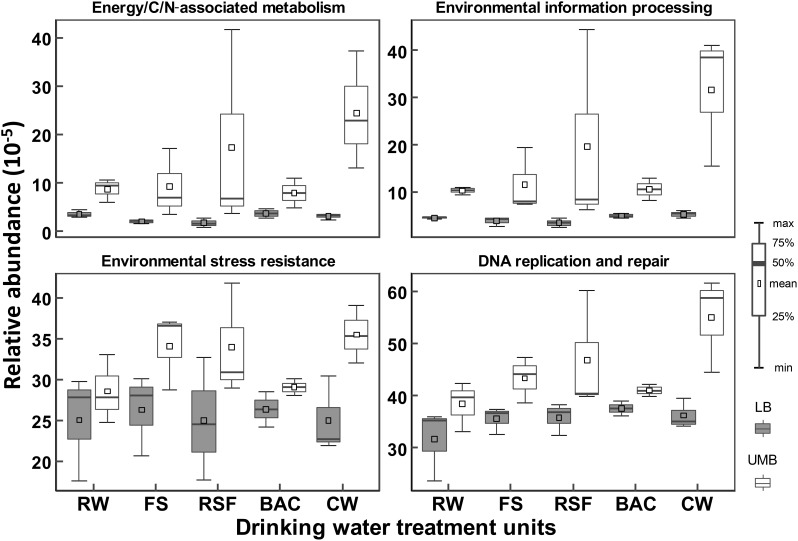
Relative abundances of inferred enzyme-encoding genes between LB and UMB in the treatment units of a DWTP. The inferred enzyme-encoding genes involved in the analysis are summarized in Supplementary Table S1.

Procrustes analysis shows that functional and metabolic capacities predicted from 16S rRNA sequencing were significantly different between LB and UMB communities (*M*^2^ = 0.73, *P* < 0.05) (Supplementary Figure S5). These significantly different genes encoding key enzymes could be classified into the following functional/metabolic categories: energy/C/N-associated metabolism, environmental information processing, environmental stress resistance and DNA replication and repair. For detailed KEGG ortholog reference profiles see Supplementary Table S1. Overall, the relative abundances of such specific predicted genes were higher in UMB than in LB (ANOVA, *P* < 0.01) (Figure 7). In addition, the higher relative abundance of such enzyme-encoding genes in UMB increased along the treatment process in the DWTP.

## Discussion

### Variance of Bacterial Cell Density and Community

In this study, we profiled the bacterial density, community composition and structure of LB and UMB in a full-scale DWTP using FCM and 0.22/0.1-μm filtration paired with high-throughput sequencing approaches. Within each individual sampling batch, the LB and UMB communities were significantly different (Figures 1, 2). Size-separation (LB vs. UMB) had an important effect on the variation of bacterial community. Filtration via the 0.2-μm pore-size enables the selection of bacteria with a sufficiently small cell structure (Wang et al., 2008a,b) and small genome (Brown et al., 2015). Luef et al. (2015) found that the bacterial community composition of a post-0.2-μm filtrate was remarkably different to that retained on the 0.2-μm filter in ground water. Besides the 0.2-μm size-separation, in nature, such size-fractionated specific distributions of community composition were also evident in 0.4-μm (Proctor et al., 2018) and 1.6-μm (Ganesh et al., 2014) filtrates. UMB and LB were, respectively, termed as low nucleic acid (LNA) and high nucleic acid (HNA) content bacteria in FCM (Wang et al., 2009; Proctor et al., 2018), and LNA were proposed to broadly encompass the categories of UMB, ultra-small bacteria, ultramicrocells, nanobacteria, and candidate phyla radiation (CPR; Proctor et al., 2018). It was demonstrated that 0.45-μm filters can essentially separate HNA and LNA based on flow cytometric fingerprints (Wang et al., 2009). However, UMB are not necessarily equal to bacteria that can pass through a 0.45-μm filter, as LB with one small diameter can also pass through 0.45-μm filters (Wang et al., 2008a). Therefore, we chose the 0.2-μm filter to separate UMB and LB in this study.

With respect to the engineered DWTP system, treatment processes have a considerable effect on the variation of bacterial communities. The cell density of both UMB and LB reduced as the treatment process progressed except in the BAC unit (Figure 1). This pattern agrees with previous studies concerning improvements of water quality in DWTPs (Zhang et al., 2017; Oh et al., 2018). As shown in Figures 2B, 6, the variation in relative abundance of different bacteria in the DWTP suggested that the drinking water treatment processes could impact bacterial communities in different ways. For example, flocculation-sedimentation and sand filtration can physically remove a considerable amount of microorganisms (Li C. et al., 2017; Hou et al., 2018); meanwhile, microorganisms that can preferentially attach to filter media surfaces would not be released with effluents (Pinto et al., 2012). The disinfection process has differential selection pressure for resistant bacteria (Ramseier et al., 2011; Gomez-Alvarez et al., 2012; Chiao et al., 2014; Inkinen et al., 2018). It was reported that the kinetics of cytomembrane damage of LB was much faster than that of UMB during chlorine dioxide and permanganate treatments (Ramseier et al., 2011). Similarly, previous studies also found that the UMB genera *Sphingomonas, Methylobacterium*, and *Legionella*, which occurred in the present study, were more abundant in the free chlorine and monochloramine disinfection effluents (Gomez-Alvarez et al., 2012; Wang et al., 2013).

Contrary to the reduction in previous treatment units, bacterial cell density and diversity in the BAC effluent increased to levels comparable to those in the RW. An explanation might be that ozonation placed before the BAC treatment unit could oxidize natural organic matter into low-molecular-weight dissolved organic carbon (DOC). This process results in very little reduction of DOC under low dose ozonation but a remarkable increase of the biodegradable DOC, namely microbial assimilable organic carbon (Velten et al., 2011; Basu et al., 2016; Besmer and Hammes, 2016). Furthermore, owing to the high specific surface area, adsorption capacity and bioavailable substrates, BAC could harbor a more dense and diverse microbiome than sand filter media (Basu et al., 2016). Consequently, with biomass accumulation and shear forces exerted by hydraulic shocks, biofilms would detach from and pass through the BAC filter media, thus increasing cell density in the BAC effluent (Boon et al., 2011; Besmer and Hammes, 2016). Nevertheless, a diverse microbial biofilm community that colonizes in BAC can contribute to improving water treatment efficiency through the metabolism of nutrients and organics (Li D. et al., 2017).

In strong contrast to the reduction of cell density and diversity, the proportion of UMB in total bacteria community increased as the treatment process progressed (Figure 1). This suggested that UMB were more persistent than LB against the treatment processes in DWTPs. The reasons for this might be that UMB could penetrate the filter media more easily than LB due to the ultra-small cell size. Moreover, the multi-stress resistance mechanisms possessed by UMB, e.g., DNA repair mechanisms, superoxide dismutase, peroxide stress response regulator and drug efflux transporter pump (Figure 7 and Supplementary Table S1), could protect UMB against the disinfection treatment process (Eguchi et al., 1996; Cavicchioli and Ostrowski, 2003; Ramseier et al., 2011).

### Occurrence of LB and UMB Indicator Taxa

Caution should be exercised when using filter-size separation of UMB since a fraction of UMB can be captured above the surface of membrane filters due to matrix effects at the beginning of filtration (Wang et al., 2008a). In addition, LB with a small diameter in one dimension can also pass through the 0.22-μm filter pore (Wang et al., 2008a; Proctor et al., 2018). Therefore, LB and UMB were not exclusively present on 0.22-μm and 0.1-μm filters, respectively, and there might be overlapping OTUs present in both groups of a filter pair (Figure 2B). To overcome such imperfections caused by filtration and to compare microbial community differentiation, the LEfSe algorithm was employed to determine specific indicator taxa for UMB and LB.

Phylogenetic classification of indicator taxa confirmed previous studies that, the LB and UMB clustering was not entirely consistent and exclusive at the phyla level (Proctor et al., 2018), and that bacterial cell size had considerable variability within a phylum (Stepanauskas et al., 2017). In spite of this, in the present study, some UMB were still exclusively assigned to CPR and symbiont phyla such as *Parcubacteria* (OD1), *Saccharibacteria* (TM7), and *Elusimicrobia* (Figures 3, 4), consistent with several prior studies showing that most CPR bacteria had ultra-small cells (Brown et al., 2015; Luef et al., 2015; Proctor et al., 2018). In addition, the data further proved the prior knowledge that the ultra-small size of UMB is an evolutionarily conserved characteristic, rather than a temporary physiological state, e.g., a starved or inactive state (Luef et al., 2015; Proctor et al., 2018).

At the genera level, only some UMB genera identified in this study, such as *Anoxybacillus* and *Stenotrophomonas*, have been previously reported as UMB. The genus *Anoxybacillus* has the smallest genomes (2.6–2.9 Mb) in the family *Bacillaceae*, and the presence of adaptive genes enable *Anoxybacillus* to survive under conditions of heat, UV irradiation, and extreme pH (Goh et al., 2014). The UMB genus *Stenotrophomonas* has been detected in natural waterborne environments (Sundaram et al., 2001) and potable drinking water (Silbaq, 2009), and is regarded as an epibiont with *Bacillus subtilis* (Shorokhova et al., 2017). Other genera, including *Kinneretia, Ralstonia, Dyella, Ramlibacter, Burkholderia paraburkholderia, Pandoraea, Roseomonas*, and *Vogesella*, have been identified as UMB for the first time in this study. It is noteworthy that the genus *Sphingomonas* is dominant in both UMB (12.1%) and LB (7.9%) indicator taxa despite numerous *Sphingomonas* sp. strains being characterized as UMB, e.g., *Sphingopyxis alaskensis* (formerly *Sphingomonas alaskensis*) (Eguchi et al., 1996; Cavicchioli et al., 2003). This phenomenon could be due to the intrinsically remarkable variability in cell size of *Sphingomonas*. Therefore, further characterization is required to explain why such different physical characteristics affiliated with a phylogenetically akin lineage (Proctor et al., 2018). The genus *Brevundimonas* also overlapped between LB and UMB indicator taxa, in which *rod-shaped Brevundimonas diminuta* was used as a standard bacterium for 0.2-μm filter-testing.

Significant positive correlation indicated higher intra-taxa co-occurrence within UMB as well as LB. As shown in Figure 5, the positive correlation between *Planctomycetes* and *Methylocystaceae*, consistent with a previous study that members of *Planctomycetes* were involved in methanogenesis and methane oxidation processes (Chistoserdova et al., 2004), might suggest cooperative relations between *Planctomycetes* and *Methylobacterium*/*Methylocystaceae*. Interestingly, the CPR bacteria, including *Parcubacteria, Dependentiae* and *Elusimicrobia*, co-occurred strongly and significantly. These non-random co-occurrence patterns revealed that phylogenetically distant UMB bacteria can share similar ecological niches, or possess cooperative relations, and such high connectedness most likely resulted from symbiotic interactions, such as mutualism, commensalism, or parasitism (Nelson and Stegen, 2015; Yeoh et al., 2016).

Natural selective pressures are likely to minimize cell complexity through optimizing bacteria metabolic burden and concomitant loss of involved genes (Giovannoni et al., 2014). The simplicity of the adaptive genome pares down the unusual growth nutrient requirement for DNA synthesis and cell growth, and provides a selective advantage under resource-limited environments (Giovannoni et al., 2005). However, such reductive evolution in streamlined metabolism also leads to the absence of some common regulatory mechanisms (Giovannoni et al., 2014). Consequently, the streamlined UMB lack numerous biosynthetic pathways for producing all essential cellular building blocks, e.g., amino acids and nucleotides, and instead depend on metabolic resources from other microorganisms, thereby living a symbiotic or parasitic lifestyle (McLean et al., 2013; Brown et al., 2015; Luef et al., 2015). For example, *Parcubacteria* (OD1) could pass through a 0.2-μm filter (Luef et al., 2015) and have a very small genome size (<1 Mb), with severely reduced metabolic functions, e.g., ATP, cofactors, amino acids, nucleotides, and fatty acids synthase (Nelson and Stegen, 2015; Suzuki et al., 2017). *Dependentiae* (TM6) was globally distributed parasitism and endosymbiosis, was associated with small genomes (1.0–1.5 Mb), and usually lacked complete biosynthesis pathways for most amino acids, lipids, and nucleotides (McLean et al., 2013; Yeoh et al., 2016). The novel phylum *Elusimicrobia*, with a diameter range of 0.15–0.30 μm, was also reported to be an intracellular symbiont (Geissinger et al., 2009; Zheng et al., 2016). In short, the streamlined genome is connected to UMB following a symbiotic or parasitic lifestyle, which then leads to the observed high connectedness as inferred above, i.e., non-random intra-taxa co-occurrence pattern within UMB.

### Predicted Functional Traits of UMB

The higher degree of specific physiological functions might contribute to adaptation of UMB to a multitude of changes in the environments, such as nutrient limitation, reactive oxygen and nitrogen species, membrane damage, elevated temperature, ribosome disruption, symbiotic interactions, and antibiotic pressure (Groisman, 2016; Zhang et al., 2017). As shown in Figure 7, the higher relative abundance of environmental information processing pathways, e.g., two-component regulatory system (Supplementary Table S1), enable UMB to respond quickly to stress conditions (Giovannoni et al., 2005; Groisman, 2016), and environmental stress resistance mechanisms (e.g., *SOD1, Fur* and *Uve1 gene* Supplementary Table S1) can protect UMB against UV and hydrogen peroxide stress (Yonemasu et al., 1997; Gomez-Alvarez et al., 2012). It was reported that *Deinococcus radiodurans*, from the phylum *Deinococcus–Thermus*, was capable of robust DNA replication and repair processes (Slade et al., 2009), and could strongly resist extreme stresses including γ-ray/UV radiation, oxidation, desiccation and high temperature (Tian and Hua, 2010). Furthermore, consistent with previous studies that the LD12 UMB lineage in *α-Proteobacteria* exhibited a higher uptake of glutamine and glutamate (Salcher et al., 2011) and UMB could efficiently utilize scarce nutrients (Giovannoni et al., 2005, 2014; Williams et al., 2009; Zhang et al., 2017), the prediction data indicated that genus with functions related to energy, carbohydrate, nucleotide and amino acid metabolism were more abundant in UMB compared with those in LB (Figure 7 and Supplementary Table S1). Altogether, these specific physiological functions likely resulted in the higher resistance of UMB to drinking water treatment processes in comparison to LB.

In summary, the current study profiled bacterial cell density, community structure and potential functions of UMB and LB in a full-scale DWTP. The data confirmed the hypothesis that UMB are more recalcitrant to drinking water treatment processes than LB despite being less diverse, and that the ultra-small structure of UMB is a naturally conserved phylogenetic trait as shown by their exclusivity at a high taxonomic level (i.e., phylum). Furthermore, the results indicated that biological activated carbon could facilitate bacterial growth, and community structure and predicted functions between UMB and LB were significantly different in DWTP. The UMB indicator taxa mainly belonged to α/β/γ-*Proteobacteria, Deinococcus–Thermus, Firmicutes, Acidobacteria*, and *Dependentiae*. In addition, the streamlined genomes is likely connected to UMB, such as CPR bacteria, following a symbiotic or parasitic lifestyle, which then results in the non-random intra-taxa co-occurrence patterns. Finally, the higher specific physiological functions, including environmental information processing and DNA replication and repair, could explain the higher resistance of UMB to drinking water treatment processes in comparison to LB.

## Author Contributions

JL, XL, and BL conceived and designed the experiments and wrote the paper. JL and RZ performed the experiments. JL, JZ, GZ, and KY analyzed the data.

## Conflict of Interest Statement

The authors declare that the research was conducted in the absence of any commercial or financial relationships that could be construed as a potential conflict of interest.
