# Supplementary material for: Occurrence and Fate of Ultramicrobacteria in a Full-Scale Drinking Water Treatment Plant
Source: Front Microbiol. 2018 Dec 5;9:2922. doi: 10.3389/fmicb.2018.02922 (PMC6290093; doi:10.3389/fmicb.2018.02922)
Supplement: Supplementary file 1 [file Table_1.docx]

**Supplementary Information for**

**Occurrence and fate of ultramicrobacteria in a full-scale drinking water treatment plant**

**Jie Liu^1^, Renxin Zhao^1^, Jiayu Zhang^1^, Guijuan Zhang^1^, Ke Yu^2^, Xiaoyan Li^1^*, Bing Li^1^***

^1^ Guangdong Provincial Engineering Research Center for Urban Water Recycling and Environmental Safety, Graduate School at Shenzhen, Tsinghua University, China

^2^ School of Environment and Energy, Graduate School, Peking University, China

*** Correspondence:**

Bing Li

[bingli@sz.tsinghua.edu.cn](mailto:bingli@sz.tsinghua.edu.cn);

Xiaoyan Li

lixiaoyan@sz.tsinghua.edu.cn;

**List of figures**

**Figure S1** Rarefaction curves for all samples.

**Figure S2** (A) Shared OTUs between LB and UMB. (B) Shared OTUs of LB in different treatment units of the DWTP. (C) Shared OTUs of UMB in different treatment units of the DWTP.

**Figure S3** Indicator taxa OTUs of LB and UMB at the phylum level. Phyla with relative abundances < 0.1% are defined as rare phyla. *P*-value is the statistical significance alpha value based on Kruskal-Wallis test in the LEfSe. The OTUs were ordered according to their taxonomy at the phylum level and then their relative abundance.

**Figure S4** Changes in total relative abundance of LB and UMB indicator taxa in different treatment units of the DWTP.

**Figure S5** Procrustes analysis of functional and metabolic capabilities between UMB and LB microbial communities.

**List of table**

**Table S1** KEGG (KO) orthologue reference profiles used to calculate the predicted relative abundance of genes within functional or metabolic type. All data were extracted from the KEGG database [www.genome.jp/kegg/](http://www.genome.jp/kegg/).

**Figure S1** Rarefaction curves for all samples.

**Figure S2** (A) Shared OTUs between LB and UMB. (B) Shared OTUs of LB in different treatment units of the DWTP. (C) Shared OTUs of UMB in different treatment units of the DWTP.

**Figure S3** Indicator taxa OTUs of LB and UMB at the phylum level. Phyla with relative abundances < 0.1% are defined as rare phyla. *P*-value is the statistical significance alpha value based on Kruskal-Wallis test in the LEfSe. The OTUs were ordered according to their taxonomy at the phylum level and then their relative abundance.

**Figure S4** Changes in total relative abundance of LB and UMB indicator taxa OTUs in different treatment units of the DWTP.

**Figure S5** Procrustes analysis of functional and metabolic capabilities between UMB and LB microbial communities.

**Table S1** KEGG orthologue (KO) reference profiles used to calculate the predicted relative abundance of genes within functional or metabolic type. All data were extracted from the KEGG database www.genome.jp/kegg/.

| **Type** | **Subtype** | **KO** | **Functional or metabolic capabilities** |
| --- | --- | --- | --- |
| Energy/C/N associated metabolism | Amino acid metabolism | K00491 | nitric-oxide synthase, bacterial [EC:1.14.13.39] |
|  | Carbohydrate metabolism | K00929 | butyrate kinase [EC:2.7.2.7] |
|  |  | K01200 | pullulanase [EC:3.2.1.41] |
|  | Energy metabolism | K00260 | glutamate dehydrogenase [EC:1.4.1.2] |
|  |  | K02826 | cytochrome aa3-600 menaquinol oxidase subunit II [EC:1.10.3.12] |
|  |  | K02827 | cytochrome aa3-600 menaquinol oxidase subunit I [EC:1.10.3.12] |
|  |  | K02828 | cytochrome aa3-600 menaquinol oxidase subunit III [EC:1.10.3.12] |
|  |  | K02829 | cytochrome aa3-600 menaquinol oxidase subunit IV [EC:1.10.3.12] |
|  | Glycan biosynthesis and metabolism | K01227 | mannosyl-glycoprotein endo-beta-N-acetylglucosaminidase [EC:3.2.1.96] |
|  | Nucleotide and amino acid metabolism | K05822 | tetrahydrodipicolinate N-acetyltransferase [EC:2.3.1.89] |
|  |  | K05823 | N-acetyldiaminopimelate deacetylase [EC:3.5.1.47] |
|  |  | K06209 | chorismate mutase [EC:5.4.99.5] |
|  |  | K13853 | 3-deoxy-7-phosphoheptulonate synthase / chorismate mutase [EC:2.5.1.54 5.4.99.5] |
|  | Nucleotide metabolism | K03816 | xanthine phosphoribosyltransferase [EC:2.4.2.22] |
|  |  | K07816 | putative GTP pyrophosphokinase [EC:2.7.6.5] |
|  |  | K15518 | deoxyguanosine kinase [EC:2.7.1.113] |
|  |  | K15519 | deoxyadenosine/deoxycytidine kinase [EC:2.7.1.76 2.7.1.74] |
| Environmental Information Processing | Competence related DNA transformation transporter | K02236 | leader peptidase (prepilin peptidase) / N-methyltransferase [EC:3.4.23.43 2.1.1.-] |
|  |  | K02240 | competence protein ComFA |
|  |  | K02243 | competence protein ComGA |
|  |  | K02244 | competence protein ComGB |
|  |  | K02245 | competence protein ComGC |
|  |  | K02246 | competence protein ComGD |
|  |  | K02248 | competence protein ComGF |
|  |  | K02250 | competence protein ComK |
|  | Drug resistance | K02547 | methicillin resistance protein |
|  |  | K03740 | D-alanine transfer protein |
|  |  | K12553 | penicillin-binding protein 3 [EC:3.4.-.-] |
|  | Two component regulatory system | K02490 | two-component system, response regulator, stage 0 sporulation protein F |
|  |  | K07704 | two-component system, LytT family, sensor histidine kinase LytS [EC:2.7.13.3] |
|  |  | K07775 | two-component system, OmpR family, response regulator ResD |
|  |  | K11617 | two-component system, NarL family, sensor histidine kinase LiaS [EC:2.7.13.3] |
|  |  | K11629 | two-component system, OmpR family, bacitracin resistance sensor histidine kinase BceS [EC:2.7.13.3] |
|  |  | K11630 | two-component system, OmpR family, bacitracin resistance response regulator BceR |
|  |  | K14988 | two-component system, NarL family, secretion system sensor histidine kinase SalK |
|  |  | K14989 | two-component system, NarL family, secretion system response regulator SalR |
|  | ABC 2 type and other transporters | K09692 | teichoic acid transport system permease protein |
|  |  | K09693 | teichoic acid transport system ATP-binding protein [EC:3.6.3.40] |
|  |  | K11631 | bacitracin transport system ATP-binding protein |
|  |  | K11632 | bacitracin transport system permease protein |
|  | Drug efflux transporter pump | K08153 | MFS transporter, DHA1 family, multidrug resistance protein |
|  | Phosphate and amino acid transporters | K10039 | putative glutamine transport system substrate-binding protein |
|  |  | K10040 | putative glutamine transport system permease protein |
|  |  | K10041 | putative glutamine transport system ATP-binding protein [EC:3.6.3.-] |
| Environmental stress resistance | Environmental stress resistance | K04565 | Cu/Zn superoxide dismutase [EC:1.15.1.1] |
|  |  | K07233 | copper resistance protein B |
|  |  | K07570 | general stress protein 13 |
|  |  | K09825 | Fur family transcriptional regulator, peroxide stress response regulator |
|  |  | K13281 | UV DNA damage endonuclease [EC:3.-.-.-] |
|  |  | K13955 | zinc-binding alcohol dehydrogenase/oxidoreductase |
| DNA replication and repair | DNA replication and repair | K02086 | DNA replication protein |
|  |  | K03346 | replication initiation and membrane attachment protein |
|  |  | K03700 | recombination protein U |
|  |  | K03763 | DNA polymerase III subunit alpha, Gram-positive type [EC:2.7.7.7] |
|  |  | K11144 | primosomal protein DnaI |
|  |  | K13531 | methylated-DNA-[protein]-cysteine S-methyltransferase [EC:2.1.1.63] |
|  |  | K03630 | DNA repair protein RadC |
|  |  | K04483 | DNA repair protein RadA |
|  |  | K04484 | DNA repair protein RadB |
